# Acute Spinal Cord Injury Due to Epidural Lipomatosis Without Osseous Injury

**DOI:** 10.7759/cureus.25212

**Published:** 2022-05-22

**Authors:** Luke Mugge, Danielle D Dang, John Dang, James Leiphart

**Affiliations:** 1 Neurological Surgery, Inova Neuroscience and Spine Institute, Falls Church, USA; 2 Neurological Surgery, Inova Fairfax Medical Campus, Falls Church, USA; 3 Internal Medicine, Walter Reed National Military Medical Center, Bethesda, USA; 4 Neurosurgery, Inova Neuroscience Institute, Falls Church, USA

**Keywords:** asia a, trauma, obesity, spinal cord injury, epidural lipomatosis

## Abstract

Spinal epidural lipomatosis (SEL) is a common pathology of the lumbar spine. While the natural history is not well understood, there is a strong association with metabolic syndrome and endocrine dysfunction. Clinical presentation typically involves slow, progressive onset of radicular and myelopathic symptoms. Treatment primarily consists of weight loss, while surgery is reserved for refractory cases or acute cauda equina syndrome. We present a case of acute spinal cord injury (SCI) after trauma with underlying SEL in the cervicothoracic spine. Additionally, a literature review using a MEDLINE search of the English literature through April 2020 following the Preferred Reporting Items for Systematic Reviews and Meta-Analyses (PRISMA) guidelines was performed to identify all documented cases of acute spinal cord injury with underlying SEL.

A 72-year-old obese male with insulin-dependent diabetes mellitus presented with subacute bilateral lower extremity weakness after a fall with a flank injury three days prior to evaluation. Within hours of admission, the patient acutely progressed to paraplegia and sensory loss below the T6 level consistent with an ASIA (American Spinal Injury Association) A spinal cord injury. No fracture or dislocation was identified on CT imaging. MRI of the thoracic spine revealed spinal cord compression secondary to extensive posterior epidural lipomatosis with resultant anterior displacement of the thecal sac. The patient underwent emergent T2-T9 laminectomy for decompression. Post-operatively, the patient regained sensation below the level of injury. A review of the literature reviewed no published articles on cases of complete spinal cord injury secondary to underlying SEL without associated fracture. Finally, we present the first report of an acute spinal cord injury in the setting of SEL without fracture. Our case demonstrates that SEL outside the lumbar spine confers increased risk for SCI following trauma. Patients with cervicothoracic SEL may require close neurological observation and timely surgical decompression.

## Introduction

Spinal epidural lipomatosis (SEL) is a condition that involves the deposition of fat within the epidural space, compromising canal diameter and possibly causing cord compression. SEL is present in approximately 2.4% of the population with 1.1% of these being symptomatic [1,2]. Obesity is a major contributing factor to the development of SEL [3]. Other predisposing conditions include thyroid disease, Cushing’s disease, steroid use, and others [4,5]. In terms of presentation, SEL typically presents concomitantly with other degenerative spine pathology. Of the patients who present with spinal stenosis, 6% will also have concurrent SEL [6]. SEL can contribute to or cause significant myelopathy secondary to stenosis and cord compression [7].

Spinal cord injury (SCI) is a much more common condition, affecting thousands of patients per year [8]. Motor vehicle accidents represent the most common causal factor and men are generally affected four times as often as women [9]. The American Spinal Cord Injury Association (ASIA) classification system remains the gold standard for the evaluation and grading of injury. Here, the extent of the injury is based on the neurological examination 24 hours after presentation [10].

We describe a case of a 72-year-old who presented with an ASIA A SCI found to have significant SEL on pre-operative MRI. This case was unique in that the patient presented in a delayed fashion and did not sustain a structural spinal injury. Despite rapid decompression via laminectomy, the patient only recovered lower extremity sensation. This is the first case to report SEL as the predominant causal factor of an ASIA A SCI from an otherwise benign injury.

## Case presentation

Case description

Clinical Presentation

**Figure 1:**
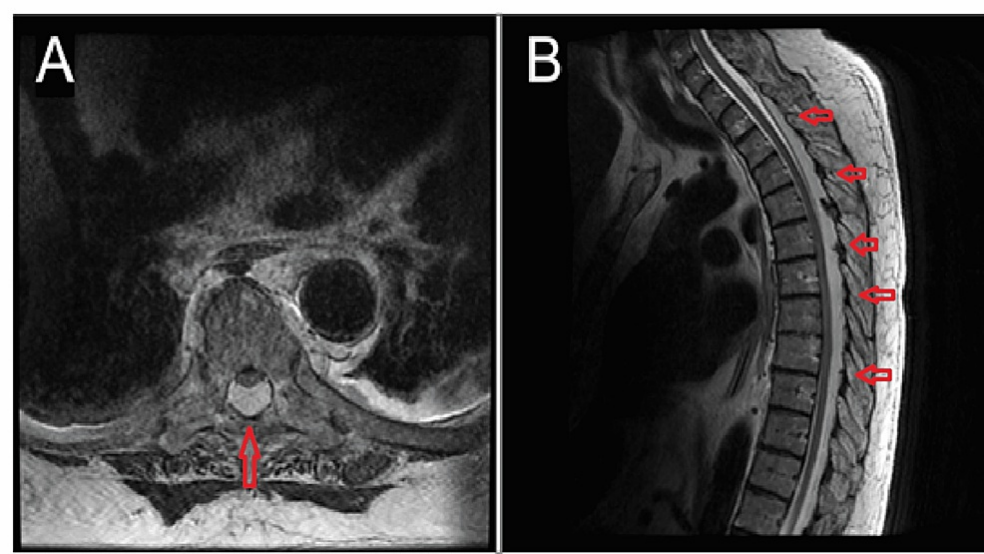
Pre-operative T2-weighted MRI revealing significant epidural lipomatosis with anterior thecal sac displacement and cord compression. A) axial view, B) sagittal view

A 72-year-old male with a past medical history of multiple myeloma, Sweet’s syndrome, hyperlipidemia, type II diabetes mellitus, and obesity (BMI: 35) presented in the emergency department with lower extremity weakness. The patient had been on prednisone for Sweet's syndrome, a rare skin condition, for several years prior to presentation. Four days prior, the patient suffered a mechanical fall from standing, without hitting his head or having any resulting sensory/motor changes immediately after the fall. The evening prior to presentation, the patient began to experience loss of sensation and weakness in his bilateral lower extremities. He presented to the hospital for evaluation. On exam, he was 0 out of 5 strength in his legs with a T8 sensory level consistent with an ASIA A SCI. He lacked hyperreflexia or clonus. There was no rectal tone and no bulbocavernosus reflex which suggested the possibility of spinal shock. MRI of the thoracic spine demonstrated prominent epidural lipomatosis spanning from C7-T12 with anterior spinal cord displacement (Figure 1).

Intervention

The patient was taken urgently to the operating room. After endotracheal intubation, the patient was positioned prone. An incision was made and the spine was exposed using sub-periosteal dissection from T4 to T7. The levels were confirmed with fluoroscopy. The spinous processes were removed along with the lamina. Immediately after, a significant amount of epidural fat was observed along the dorsum of the spine within the epidural space. Pathological analysis confirmed that this was indeed adipose tissue. The remaining epidural lipomatosis was resected. The incision and laminectomy were then extended from T2 rostrally down to T9 caudally in order to completely decompress the spinal cord. The patient tolerated the procedure well without immediate complications.

Post-operative Course

**Figure 2:**
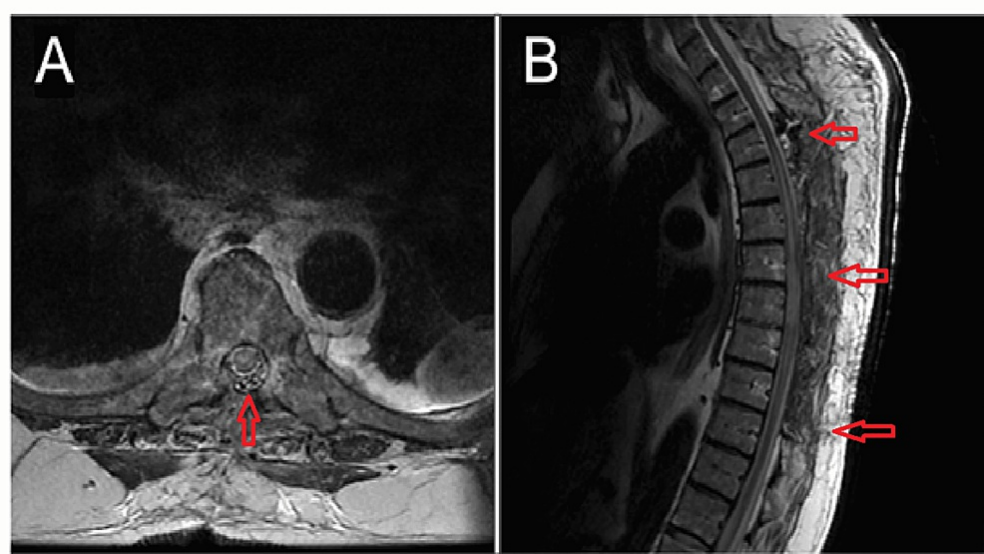
Postoperative T2-weighted MRI demonstrating post-surgical decompression and absence of thecal sac displacement. A) axial view, B) sagittal view

In the recovery unit, the patient was still noted to have a T8 sensory level and remained 0 out of 5 in strength in bilateral lower extremities. The patient was taken to the intensive care unit (ICU) where his mean arterial pressure (MAP) were held above 85 for a total of 72 hours. Repeat MRI of the thoracic spine demonstrated post-surgical changes with laminectomies from T2 to T9 with the resolution of stenosis or spinal cord compression. There was interval development of cord signal change consistent with edema spanning from T3 to T10 (Figure 2).

On postoperative day 4, the patient regained sensation in his lower extremities but remained 0 out of 5 in strength consistent with an ASIA B SCI. The remainder of the patient’s hospital stay was unremarkable and the patient was discharged to a rehab facility.

Literature review

Methods

An extensive search of the literature was completed in order to identify published literature regarding the incidence of SCI in the setting of SEL. A review of the MEDLINE data bank was completed according to “The Preferred Reporting Items for Systematic Reviews and Meta-Analyses (PRISMA)” [11]. Published literature through April 2022 was reviewed. Only articles in English were considered for review. Search terms were as follows: “epidural lipomatosis AND spinal cord injury”, “epidural lipomatosis AND spine injury”, and “epidural lipomatosis AND trauma”. Articles were excluded if they did not discuss SEL in any form or if there was no associated trauma directly or indirectly related or attributed to known underlying SEL.

Results

A total of 162 articles were identified in this way. After screening via review of titles, abstracts, and full-length articles, no studies or reports were identified which describe an SCI in the context of SEL without an associated osteosis injury. Thus, we believe that this is the first report of an SCI attributed to SEL in the context of a low-velocity trauma without an associated osteosis injury.

## Discussion

We present a case of SCI in the context of SEL without spinal fracture or dislocation after a minor, low-velocity trauma where the patient presented in a delayed fashion. More significantly, this is the first case that describes an SCI with SEL as the sole significant image finding. SEL is predominantly indolent and slowly progressive. However, this case highlights its potential to be involved in trauma in catastrophic ways. We hypothesize that SEL played a significant role in predisposing our patient to SCI by compromising normal canal diameter, causing spinal cord compression, and permitting direct cord contusion from otherwise indolent trauma.

SEL is well documented as a contributor to diminished spinal canal diameter, which predisposes patients to spinal pathologies. Given the slowly progressive nature of SEL, it classically presents with symptoms of myelopathy from central compression [4]. However, SEL has resulted in Cauda equina syndrome (CES) a sufficient number of times that studies indicate that SEL should be suspected when there is no obviously identifiable disc fragment or alternative pathology [12,13]. Even in pediatric populations, SEL has resulted from hormonal replacement therapy and has led to spastic paraparesis in cases of chronic steroid use [14,15]. In our case, chronic steroid use for a rare skin condition contributed to the progression of SEL, predisposing the patient to neurological injury. 

Not all cases of SEL are clinically significant. Upwards of 1 in 40 patients have SEL with 23% having no symptoms [16]. Nonetheless, its occasional contribution to neurological deficits and, as in our case, to devastating injury should raise concern when it is encountered incidentally. Regarding treatment, weight loss is a well-documented strategy, which can lead to the dissipation of SEL and complete amelioration of symptoms [17]. For cases where weight loss is unsuccessful or, as with our case, where significant neurological deficits are present or impending, surgical decompression and evacuation are preferred. Ferlic et al. demonstrated a meaningful improvement in pain control with surgical decompression [18].

Taken together, SEL, while commonly seen, rarely has significant pathological implications. It is predominantly observed incidentally or as a component of degenerative spine disease. As with most degenerative or chronic pathologies, there is a significant role for conservative management, i.e., weight loss. Surgery should be reserved for severe cases unresponsive to medical management or when neurological deficits are present. However no randomized controlled trials have examined this recommendation, and future research is warranted to determine the most appropriate therapeutic approach. Given our findings, we propose that physicians should counsel patients about the risks of neurological injury when there is a known metabolic syndrome or when chronic steroids are being used given that both have the potential for SEL development. Despite the slow progression, decreased canal diameter is a result of SEL and places patients at significant risk for neurological injury, even without associated fracture or ligamentous injury in the setting of trauma.

## Conclusions

We present a rare case of a patient with a significant SCI after an otherwise indolent trauma, who was noted to have profound SEL but specifically lacked spinal fracture or dislocation. This is the first such reported case to our knowledge. While SEL is commonly regarded as a slowly developing disease process, this case highlights a significant decrease in spinal diameter caused by SEL, which placed the patient at risk for neurological injury even after relatively minor trauma. There is a need for clinicians to counsel patients about the presence of SEL and the relative risk that it poses, encouraging appropriate management in order to avoid more devastating outcomes.
